# Supplementary material for: Dietary Supplementation with Omega-3 Polyunsaturated Fatty Acids Reduces Opioid-Seeking Behaviors and Alters the Gut Microbiome
Source: Nutrients. 2019 Aug 14;11(8):1900. doi: 10.3390/nu11081900 (PMC6723154; doi:10.3390/nu11081900)
Supplement: Supplementary file 1 [file nutrients-11-01900-s001.pdf]

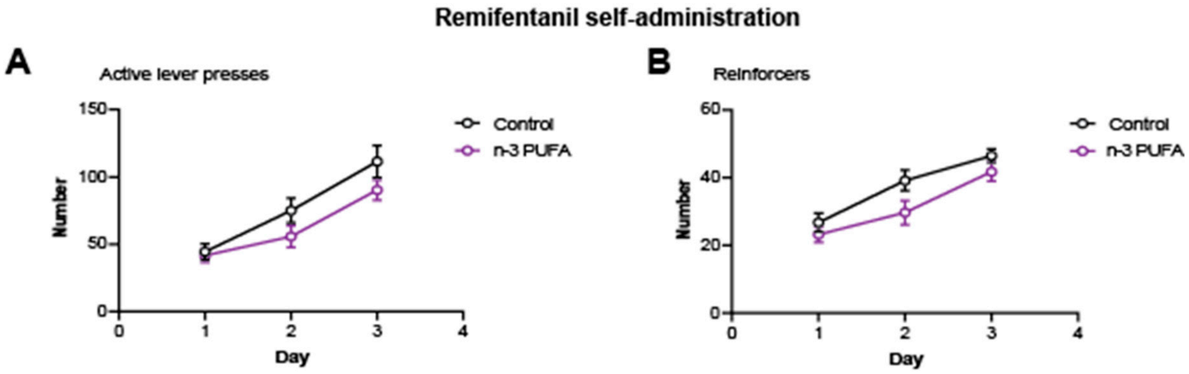

Supplemental Figure S1. The acquisition of remifentanil self-administration. (A) There was no effect of diet (control diet shown in black, n-3 PUFA diet shown in purple) in the initial acquisition of opioid self-administration using remifentanil as the RNFS when observing (A) AL presses (p=0.3) or **(B)** RNFS earned (p=0.4).
